# Mating-type locus structure affects gene expression in unidirectional mating-type switching fungi

**DOI:** 10.1093/g3journal/jkag128

**Published:** 2026-05-12

**Authors:** Frances A Lane, Brenda D Wingfield, Michael J Wingfield, P Markus Wilken

**Affiliations:** Department of Biochemistry, Genetics and Microbiology, Forestry and Agricultural Biotechnology Institute (FABI), University of Pretoria, Private Bag X20, Hatfield 0028, South Africa; Department of Biochemistry, Genetics and Microbiology, Forestry and Agricultural Biotechnology Institute (FABI), University of Pretoria, Private Bag X20, Hatfield 0028, South Africa; Department of Biochemistry, Genetics and Microbiology, Forestry and Agricultural Biotechnology Institute (FABI), University of Pretoria, Private Bag X20, Hatfield 0028, South Africa; Department of Biochemistry, Genetics and Microbiology, Forestry and Agricultural Biotechnology Institute (FABI), University of Pretoria, Private Bag X20, Hatfield 0028, South Africa

**Keywords:** homothallism, gene regulation networks, filamentous fungi, Fungal 2026

## Abstract

Fungal species are typically either fully self-fertile or self-sterile, but some filamentous ascomycetes can transition from self-fertility to self-sterility through unidirectional mating-type switching. In these fungi, the structure of the mating-type (*MAT1*) locus governs sexual behavior: MAT-2 self-fertile individuals retain both *MAT1-1* and *MAT1-2* genes, while MAT-1 self-sterile isolates lose *MAT1-2* genes during switching. A third type of isolate morphology also occurs under laboratory conditions: these are self-sterile isolates that retain both *MAT1-1* and *MAT1-2*, but are unable to switch mating type. They are commonly referred to as MAT-2 self-sterile isolates. Two of the mating-type (*MAT*) genes, one of which is deleted during switching, encode transcription factors known to regulate not only the sexual cycle but also genes unrelated to mating. To test how *MAT1* structural variations affect gene expression, we studied *Ceratocystis albifundus*, a species that switches mating type. To minimize variability caused by intraspecific genetic differences, 2 self-sterile isolates (MAT-1 and MAT-2 self-steriles) were derived from the same MAT-2 self-fertile parent, making all 3 isolates genetically identical, except at the *MAT1* locus. Comparative transcriptomic analyses revealed that the MAT-2 self-fertile, MAT-1 self-sterile, and MAT-2 self-sterile isolates all exhibited distinct expression patterns, including differences in *MAT* genes, the pheromone–receptor pathway, and other genes not directly linked to mating. The results show that *MAT1* locus structure influences gene expression more broadly than those only related to the sexual cycle.

## Introduction

Sexual reproduction in ascomycetes is controlled by a single genomic region known as the mating-type (*MAT1*) locus (Wilson et al. 2021). The gene content and overall architecture of the *MAT1* locus drive mating behavior and reproductive strategy. In its simplest form, the *MAT1* locus is defined by the *MAT1-1-1* and/or *MAT1-2-1* genes (Turgeon and Yoder 2000; Wilken et al. 2017), with both encoding transcription factors that influence gene expression, ultimately enabling the sexual cycle (Herskowitz 1989; Dyer et al. 2016; Wilson et al. 2021). The gene content and structure of the *MAT1* locus can vary across fungi, and in some cases even show variation within members of a single species (Butler 2007). This variation accounts for most of the diversity of fungal mating strategies (Yun et al. 1999; Gioti et al. 2012; Wilson et al. 2015).

Most fungi have either a heterothallic or homothallic mating strategy (Butler 2007; Debuchy et al. 2010; Bennett and Turgeon 2016). Heterothallic species are self-sterile as they require a partner of opposite mating type to complete the sexual cycle. In contrast, homothallic fungi can self-fertilize to produce sexual spores, but this ability can arise through several distinct genetic mechanisms (Dodge 1957; Perkins 1987; Glass and Smith 1994; Ni et al. 2011; Wilson et al. 2015). The most well-known of these is primary homothallism, where all the genes driving mating type are present in a single isolate (Pöggeler et al. 1997). Other mechanisms also enable self-fertility in fungi, such as mating-type switching, where genetic alterations in the *MAT1* locus drive self-fertility. While bidirectional mating-type switching is well characterized in yeasts (Haber 2012; Hanson and Wolfe 2017), unidirectional switching in filamentous ascomycetes is comparatively less well understood.

Unidirectional mating-type switching produces both self-fertile and self-sterile progeny from a single sexual event, whether by selfing or outcrossing (Perkins 1987). This behavior has only been described in a small number of species residing in 4 unrelated fungal families and 2 different classes; the *Ceratocystidaceae* (Harrington and McNew 1997), *Glomerellaceae* (Wheeler 1950), and *Hypocreaceae* (Mathieson 1952) in the *Sordariomycetes*, and the *Sclerotiniaceae* (Uhm and Fujii 1983a, 1983b) in the *Leotiomycetes*. In 2 of these species, *Chromocrea spinulosa* (*Trichoderma spinulosum*) and *Sclerotinia trifoliorum*, the ascospores that give rise to self-fertile and self-sterile strains differ in size (Wheeler 1950; Uhm and Fujii 1983a, 1983b). While this spore dimorphism is not known in any of the species that undergo unidirectional mating-type switching in the *Ceratocystidaceae* (Harrington and McNew 1997), there have been some reports that the self-fertile isolates are more pathogenic (Lee et al. 2015). This is not surprising as several studies on fungi residing in other families have shown that mating-type genes directly influence many other aspects of their biology, including vegetative growth rate, conidial structure and pathogenicity (Lockhart et al. 2005; Zhan et al. 2007; Yong et al. 2020).

**Fig. 1.**
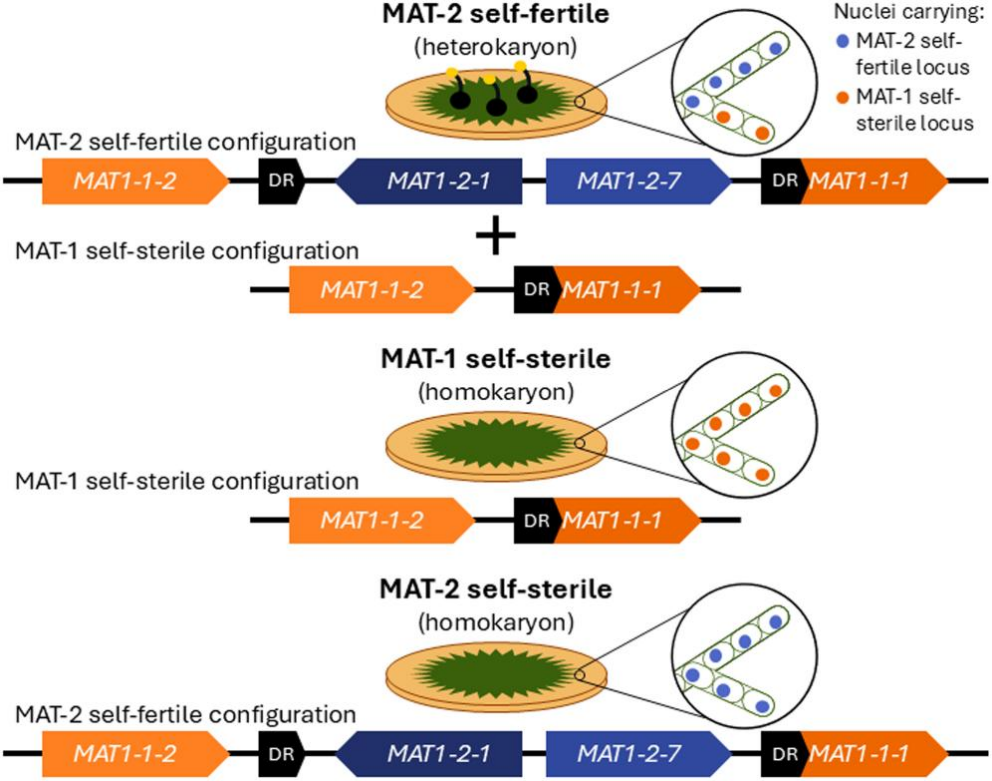
A graphic representation of the 3 isolate types that occur in *C. albifundus* with the *MAT1* locus versions that they carry. Self-fertility is indicated by a culture (in green) with an ascomata (round, black ascomatal bases with long necks and yellow spore drops). Self-sterility is indicated by a culture lacking sexual structures. Hyphae (image in black circles) are made up of cells with nuclei carrying either the self-fertile locus configuration (blue circles) or the MAT-1 self-sterile locus configuration (orange circle). The *MAT1* locus configuration present in the isolates is presented below the image of each culture. *MAT1-1* genes are indicated by orange arrows, while *MAT1-2* genes are blue. Black arrows indicate direct repeats (DR).

Recent work has highlighted key genetic mechanisms underlying unidirectional mating-type switching in fungi. Self-fertile isolates appear to exist as heterokaryons that differ only at the *MAT1* locus (Yun et al. 2017). One version of the *MAT1* locus, known as the MAT-2 self-fertile version, contains both *MAT1-1* and *MAT1-2* genes (Fig. 1), with the *MAT1-2* genes typically flanked by 2 direct repeats and inserted within the *MAT1-1-1* gene, disrupting its function (Xu et al. 2016; Yun et al. 2017; Wilken et al. 2024). The alternative, MAT-1 self-sterile version contains only *MAT1-1* genes and, when present as a homokaryon, is self-sterile (Fig. 1). Unidirectional mating-type switching produces this self-sterile form by deleting the *MAT1-2* genes and one of the flanking direct repeats (Witthuhn et al. 2000; Wilken et al. 2014), thereby restoring *MAT1-1-1* function (Wilken et al. 2024). Despite their self-sterility, these isolates retain the ability to mate and can outcross with self-fertile strains to produce viable offspring (Harrington and McNew 1997; Yun et al. 2017). These findings highlight the central role of unidirectional mating-type switching in generating both self-fertile and self-sterile isolates, ultimately enabling sexual reproduction to occur.

Interestingly, some species in the *Ceratocystidaceae*, including members of the genus *Ceratocystis*, have an unusual self-sterile morphotype (Harrington and McNew 1997). In this case, individuals are referred to as MAT-2 self-sterile isolates and have exclusively been described in laboratory strains. Amplification of locus-version-specific regions combined with amplicon size analysis (Supplementary Fig. 1) demonstrated that these isolates are homokaryotic, carrying a *MAT1* locus with both *MAT1-1* and *MAT1-2* genes in the configuration characteristic of the MAT-2 self-fertile isolates (Fig. 1; Oliveira et al. 2015; Wilken 2015; Engelbrecht and Harrington 2017; Krämer et al. 2021). This suggests that they do not undergo mating-type switching as the *MAT1-2* genes are still present. Although the cause of sterility in MAT-2 self-sterile mutants is unknown, they have been used in mating and hybridization studies where they exclusively act as a male mating partner (Johnson et al. 2005; Oliveira et al. 2015; Fourie et al. 2019).

*Ceratocystis albifundus* is emerging as a genetic model for studies on members of the genus *Ceratocystis*. Current research efforts for this species include the development of an *Agrobacterium*-mediated transformation system, a population-level genomic study, and the identification of its pheromone–receptor system (Sayari et al. 2019; van der Nest et al. 2019; Danki et al. 2024; Lane et al. 2025). The MAT-2 self-fertile, naturally occurring MAT-1 self-sterile, and laboratory-derived MAT-2 self-sterile isolates have been described (Lee et al. 2018). The *MAT1* locus of MAT-2 self-fertile isolates contains complete copies of *MAT1-1-1*, *MAT1-1-2*, *MAT1-2-1*, and *MAT1-2-7* (Lee et al. 2018; Wilken et al. 2024). During unidirectional switching, the *MAT1-2* genes are deleted, producing a MAT-1 self-sterile isolate. Although *MAT1-1-1* is structurally intact in the self-fertile morphotype, it appears separated from its promoter by the positioning of the *MAT1-2* genes. Limited RT-qPCR experiments suggest the deletion of the *MAT1-2* region joins *MAT1-1-1* to its promoter, enabling expression of the complete gene (Wilken et al. 2024). However, the expression profile of the mating-type genes and other genes involved in the sexual cycle of this fungus remains to be explained.

The ability of *Ceratocystis* species to produce both self-fertile and self-sterile versions of the mating-type locus within an otherwise uniform genetic background makes them ideal to study how *MAT1* structure influences gene expression. In these fungi, the phenotype of an isolate, whether it is self-fertile or self-sterile, is determined directly by the arrangement of the *MAT1* locus. The aim of this study was to explore how differences at this single locus influence gene expression in isolates of *C. albifundus*. As these are haploid fungi, self-fertile reproduction through haploid-selfings produces genetically identical offspring (Billiard et al. 2012). As such, the isolates used were assumed to be genetically identical except at the *MAT1* region. This made it possible to consider how self-sterile isolates, which functionally mimic heterothallic mating types, differ from each other and from a self-fertile isolate that carries both idiomorphs. By examining the transcriptomes of the isolate types, we gained insights into how *MAT* genes are expressed within different locus structures, and how variation at the *MAT1* locus alone can shape overall genome-wide gene expression.

## Materials and methods

### Annotated genome assembly for functional analysis

The genome assembly of *C. albifundus* isolate CMW 4068, a MAT-2 self-fertile isolate, was obtained from NCBI (GenBank accession GCA_002742255) and was annotated using data provided by van der Nest et al. (2019). The *MAT1*, pheromone, and pheromone–receptor loci were screened to confirm that the annotations included all genes expected in these regions (Wilken et al. 2024; Lane et al. 2025), and, where missing, they were manually added. Annotations for the direct repeats were also added to the *MAT1* locus.

The mating-type locus from the genome was edited manually to produce 2 loci that were representative of the transcriptionally active *MAT* gene structure (Wilken et al. 2024). To do this, the *MAT1* locus was located based on a tBLASTn search using the *MAT* genes described in *C. albifundus* (Wilken et al. 2024). This locus was edited by removing the complete *MAT1-2* region, which consisted of the *MAT1-2-1* and *MAT1-2-7* genes and a single direct repeat. This produced the *MAT1* locus corresponding to the MAT-1 self-sterile version with the *MAT1-1-1* gene linked to its promoter (Wilken et al. 2024). To retain the *MAT1-2* genes, the removed region was artificially included in the genome assembly as a freestanding contig. The genome assembly was functionally annotated using the OmicsBox (Bioinformatics Made Easy, BioBam Bioinformatics) Blast2GO suite, which included a Diamond BLAST followed by InterProScan, and EggNOG analyses (Gotz et al. 2008; Huerta-Cepas et al. 2016; Huerta-Cepas et al. 2017; Blum et al. 2021).

### Generation of self-sterile cultures

*C. albifundus* isolate CMW4068 was obtained from the culture collection (CMW) of the Forestry and Agricultural Biotechnology Institute (FABI) based at the University of Pretoria. This culture was grown at 25 °C on 2% malt extract agar (MEA; Biolab, Merck, Johannesburg, South Africa) supplemented with 150 mg/ml thiamine and 100 mg/ml streptomycin (TS; SIGMA, Steinheim, Germany). After approximately 2 weeks of incubation, ascomata having drops of ascospores at their apices, typical of self-fertility in this species, were observed. This isolate was designated as MAT-2 self-fertile in the study. Single ascospore isolates were generated from this culture using serial dilution as described in Krämer et al. (2021). Single ascospore cultures that did not produce ascomata after 2 weeks of incubation were treated as putatively self-sterile. These isolates were subcultured for 2 more rounds by transferring a block of agar covered with mycelium to a fresh MEA-TS plate. When these cultures remained sterile, they were further screened for the presence of one or both versions of the mating-type locus.

DNA was isolated from all potential self-sterile cultures and the corresponding self-fertile culture using a CTAB protocol (Krämer et al. 2021) and subjected to PCR screening to determine *MAT1* locus structure. Two primer sets targeting the *MAT1* locus were used to distinguish between the MAT-2 self-fertile and MAT-1 self-sterile versions, with 2 additional gene-specific primer sets included for confirmation (Supplementary Table 1; Supplementary Fig. 1). All PCR amplifications were carried out in separate 25 μl reactions consisting of 10 to 50 ng of template DNA, 1 U KAPA Taq DNA Polymerase (KAPA Biosystems, Boston, MA, United States), 1×KAPA Taq Buffer A, 0.2 mM dNTP mix, and 0.4 μM of each primer. The DNA from the MAT-2 self-fertile isolate served as a positive control as these cultures are expected to carry both versions of the *MAT1* locus and all *MAT* genes (Wilken et al. 2024). Amplification reactions were performed as described in Wilken et al. (2024) using an annealing temperature of 55 °C. PCR amplicons were visualized via gel electrophoresis, and the results were used to identify the self-sterile isolates as either MAT-1 self-sterile or MAT-2 self-sterile.

### RNA isolation and sequencing

A single isolate of each of the 3 isolate types (MAT-2 self-fertile, MAT-1 self-sterile, and MAT-2 self-sterile) was selected for further analysis. For each isolate type, 9 petri dishes containing 2% MEA-TS covered with a sterile cellophane disc (BioRad, Johannesburg, South Africa) were inoculated with a block of mycelium and incubated for 13 d in the dark at 25 °C. This timepoint was selected as the MAT-2 self-fertile isolate had produced ascomata with exuded ascospore drops, and the self-sterile isolates had grown to a size sufficient for nucleic acid extraction. For RNA extraction, the mycelium growing on the surface of the cellophane from 3 plates was pooled into a single extraction, and this was done in triplicate for the 9 cultures per isolate (resulting in 3 RNA samples for each isolate type). RNA extractions were performed using the RNeasy Plant extraction kit (Qiagen, Limburg, The Netherlands) following the manufacturer's instructions, apart from using the RLC buffer and including a DNase extraction step using a DNase-I kit (RNase-Free DNase Set, Qiagen, Limburg, The Netherlands). RNA sample quantity and quality were assessed using gel electrophoresis and a NanoDrop ND-1000 (ThermoScientific, Waltham, United States). RiboLock RNase Inhibitor (ThermoScientific, Waltham, United States) was added to each sample before storage at −80 °C.

RNA library preparation and sequencing were performed by the Agricultural Research Council Biotechnology Platform (ARC-BTP, Onderstepoort, South Africa). The library was produced using an Illumina TruSeq Stranded Total RNA kit (Illumina, San Diego, CA, United States), which includes the removal of rRNA and capturing of both coding and noncoding RNA, the quality of which was assessed using a Qubit 3.0 fluorometer (ThermoFisher Scientific, Waltham, MA, United States) and LabChip GX Touch (Perkin Elmer,Waltham, MA, United States) instrument. The RNA sequencing was completed on an Illumina HiSeqX instrument (Illumina, San Diego, CA, United States).

### Expression analysis

CLC Genomics Workbench (v. 22.0; CLC Bio, Aarhus, Denmark) was used to map the raw reads to the target genome assembly. Raw reads were trimmed using the default settings of the built-in “Trim Reads” tool, and the quality was assessed using the QC function. RNA reads of each isolate type were mapped to the modified *C. albifundus* annotated genome using the “RNA-Seq Analysis” tool. Default settings were used except for the length fraction, which was set to a minimum of 0.5.

The R studio package zFPKM (v. 1.26.0) was used to normalize the mapped reads across samples and to identify genes that were expressed relative to background gene expression (Hart et al. 2013). Fragments per kilobase of transcript per million mapped reads (FPKM) values for the coding sequence (CDS) of each gene generated by CLC Genomics Workbench was normalized using zFPKM. The mean zFPKM value across the 3 replicates for each sample type was calculated and genes with a mean zFPKM of more than or equal to −3 (as recommended by Hart et al. 2013) were considered expressed.

Differential gene expression was performed by DESeq2 (v. 1.44.0; Love et al. 2014) using filtered CDS datasets of raw counts and comparing the MAT-2 self-fertile to both self-sterile isolates separately as well as comparing the self-sterile isolates to each other. Genes that were not expressed (based on the zFPKM values) in both isolates being compared were removed to reduce background noise. The *MAT1-2-1* and *MAT1-2-7* genes were also removed in comparisons with the MAT-1 self-sterile isolates as these genes are absent in these genomes and thus would not show any expression. Principal component analyses (PCA) were performed on rlog-transformed, DESeq2-normalized count matrices that were not filtered by zFPKM expression thresholds, in order to capture global transcriptional variation across all expressed genes. In contrast, zFPKM-filtered CDS count matrices were used for differential expression analysis and for downstream visualization.

Genes were considered significantly differentially expressed between sample types if there was a DESeq2-estimated log2 fold change (log2 FC) greater than 0.58 or less than −0.58 (≈ 1.5-fold change), calculated from size-factor normalized read counts, with an adjusted *P*-value of less than 0.05 (Joubert et al. 2024). For each pairwise comparison, the top 10 genes with the highest positive log_2_ FC for each isolate in each expression comparison were selected for visualization. Heatmaps were generated using rlog-transformed normalized counts, with *Z*-score scaling applied per gene to visualize relative expression patterns across samples. Among these top upregulated genes, any described as “hypothetical” based on BLAST results were translated, and conserved domains were identified using a Pfam (Pfam-A database v. 37.2) search in CLC Main to discern possible functions for these gene products. The expression of the mating-type genes and genes involved in the pheromone/receptor pathway (Supplementary Table 2) was similarly visualized using heatmaps generated from rlog-transformed normalized counts with gene clustering. These genes were included as they are key components responsible for sexual reproduction, partner signaling, pheromone processing, and signal transduction.

Gene set enrichment analysis (GSEA; Mootha et al. 2003; Subramanian et al. 2005) was used to determine the overall pathway enrichment of each comparison. The sign(log_2_FC)×-log_10_(*P*-value) from the DESeq2 output was used as the ranking metric (Stavely et al. 2023). Gene sets were considered enriched if they had an FDR *P*-value of less than 0.25, a normalized enrichment score (NES) of more than 1 or less than −1 and a nominal (NOM) *P*-value of less than 0.05.

The mapping of the reads from the mating-type genes was further analyzed. To do this, the 3 RNA datasets for each isolate type were mapped to the original MAT-2 self-fertile genome assembly, as well as a MAT-1 self-sterile genome assembly (produced in silico by deleting the region between the direct repeats and including one such repeat). The mapping results were inspected at the *MAT1* locus for anomalies in the read distribution across the genes and different locus configurations.

## Results

### Annotated genome assembly for functional analysis

The genome assembly of the MAT-2 self-fertile isolate CMW 4068 of *C. albifundus* was obtained and annotated using data from van der Nest et al. (2019). After manually annotating the a-pheromone and *MAT1-2-7* genes, a total of 7,024 gene annotations were present in the genome assembly. Of these genes, the Blast2GO pipeline identified 2,185 as hypothetical proteins, with 1,262 of these assigned GO terms. A further 223 genes lacked homology to genes in the searched database and were not assigned a descriptive name or GO term. These genes were subsequently referred to as putative genes in this study.

The mating-type locus was identified on contig MAOA02000032 and was identical to the self-fertile *MAT1* locus version previously described (accession OR922808; Wilken et al. 2024). It contained 4 mating-type genes, *MAT1-1-2*, *MAT1-2-1*, *MAT1-2-7*, and *MAT1-1-1* (Fig. 1). Two 84 bp direct repeats, flanking the *MAT1-2* genes, were also identified. The first direct repeat was located between the *MAT1-1-2* and *MAT1-2-1* genes, while the second formed part of the 5′ end of the *MAT1-1-1* open reading frame.

A 3,348 bp region flanked by the direct repeats would have been deleted through mating-type switching. This region included *MAT1-2-1* and *MAT1-2-7*, and a single copy of the repeat. This was removed from the contig and inserted into the genome assembly as a stand-alone contig. By doing so, the self-fertile *MAT1* locus version present in the genome assembly was replaced by the self-sterile version where the *MAT1-1-1* gene is placed directly downstream of its promoter (Wilken et al. 2024). The *MAT1-2* genes were retained in a separate contig together with their immediately flanking regions to avoid disrupting any potential promoter sequences. This allowed for more accurate mapping of the expression data.

### Generation of self-sterile cultures

The *C. albifundus* (CMW 4068) culture produced abundant fertile ascomata when cultured in isolation, and was confirmed as a MAT-2 self-fertile individual. Forty single-spore colonies were derived from this isolate, of which 5 consistently lacked ascomata after 2 additional rounds of culturing, and were treated as putatively self-sterile. PCR screening showed that one isolate possessed only the MAT-1 self-sterile version of the *MAT1* locus, with no amplicons matching the MAT-2 self-fertile locus or *MAT1-2-1* gene being present. This isolate was subsequently treated as a MAT-1 self-sterile. The PCR results for the remaining 4 isolates consistently produced the expected amplicons for the *MAT1-1-2* and *MAT1-2-1* genes, as well as the MAT-2 self-fertile *MAT1* locus version. The structure of the *MAT1* locus, combined with the self-sterility, was indicative of MAT-2 self-sterile isolates. One of these was randomly chosen for the remainder of the study. The MAT-1 and MAT-2 self-sterile isolates (CMW 67508 and CMW 67509, respectively) have been deposited into the culture collection (CMW) of the Forestry and Agricultural Biotechnology Institute (FABI) at the University of Pretoria.

### RNA isolation and sequencing

The RNA extracted was of sufficient quality and quantity to produce expression data representative of each isolate type across all 3 replicates (Supplementary Table 3). For each of the 3 isolates (with 3 replicates each), RNA-seq produced an average of 37.1 million raw reads for the MAT-2 self-fertile, 44.7 million reads for the MAT-1 self-sterile, and 35.6 million reads for the MAT-2 self-sterile (deposited in the NCBI Sequence Read Archive). After quality trimming, at least 99.997% of the reads were retained, with a minimum of 88.58% of these reads mapping to the genome (in pairs and in broken pairs). This provided confidence in the subsequent expression analysis.

### Expression analysis

Of the 7,024 predicted genes in the genome, 6,495 were consistently expressed across all isolates, indicating strong transcriptome coverage and a high level of overall expression. To reduce background signal and enhance differential expression analysis, genes not expressed in both isolates for a given comparison, or absent from the MAT-1 self-sterile genome (i.e. *MAT1-2-1* and *MAT1-2-7*), were removed. This included 339 genes not expressed in any isolate, and a small number of additional genes specific to each comparison: 41 for the self-fertile vs MAT-1 self-sterile, 12 for the self-fertile vs MAT-2 self-sterile, and 53 for the MAT-1 vs MAT-2 self-sterile comparison. The extensive overlap in expressed genes suggested that there was a low risk of failing to detect expression differences.

PCA confirmed data quality, replicate consistency, and clear separation between isolate types. The PCAs revealed clustering of technical replicates for each sample, with the PC1 accounting for 58% variance between isolate types and a total variance of 86% (Supplementary Fig. 2). Based on this plot, the largest source of variance in this dataset was associated with isolate type, and no strong batch effect was linked to collection date.

**Fig. 2.**
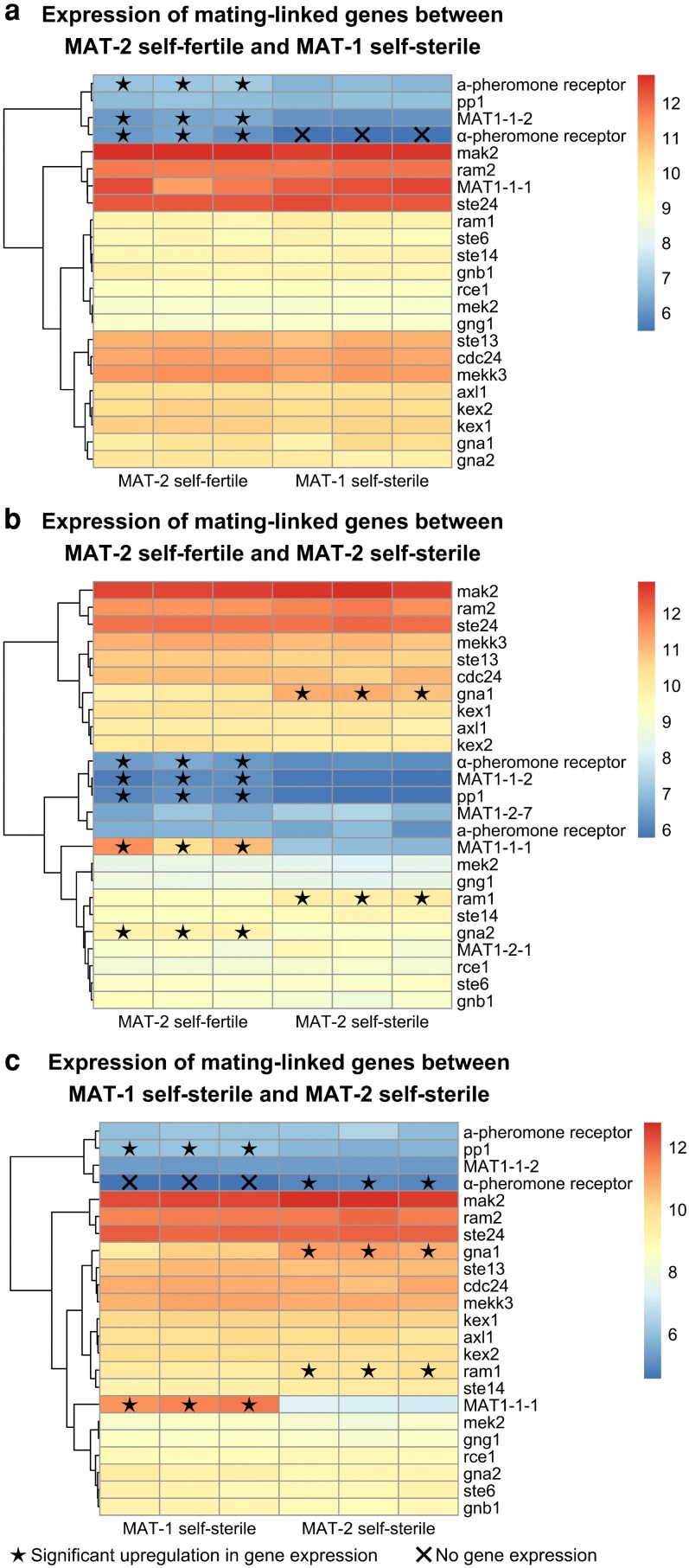
Heatmaps showing the transformed, relative expression of mating-type and mating-related genes in *C. albifundus* isolates compared between (a) MAT-2 self-fertile and MAT-1 self-sterile isolates, (b) MAT-2 self-fertile and MAT-2 self-sterile isolates, and (c) MAT-1 self-sterile and MAT-2 self-sterile isolates. Expression values represent rlog-transformed read counts generated by DESeq2 and are shown on the color scale. Warmer colors (red) indicate higher levels of expression, while cooler colors (blue) represent lower expression. Genes significantly upregulated in one isolate relative to the other are indicated by black stars (adjusted *P*-value < 0.05, |log_2_FC| > 0.58). Crosses indicate genes that were not expressed in that isolate (zFPKM ≤ −3). The 2 pheromone genes were not included because they were not expressed in any isolate. Gene clustering was performed based on expression similarity.

Genes associated with the mating-type and pheromone signaling pathways differed in their relative expression levels, with broadly similar patterns among isolates. Both secondary mating-type genes (*MAT1-1-2* and *MAT1-2-7*) and the pheromone–receptor genes were expressed at low levels (Fig. 2). In contrast, *mak2* (involved in signal transduction), as well as *ram2* and *ste24* (involved in a-pheromone post-translational modification), showed consistently high expression relative to the other mating-related genes. Notably, neither the a- nor α-pheromone genes were expressed in any isolate.

**Fig. 3.**
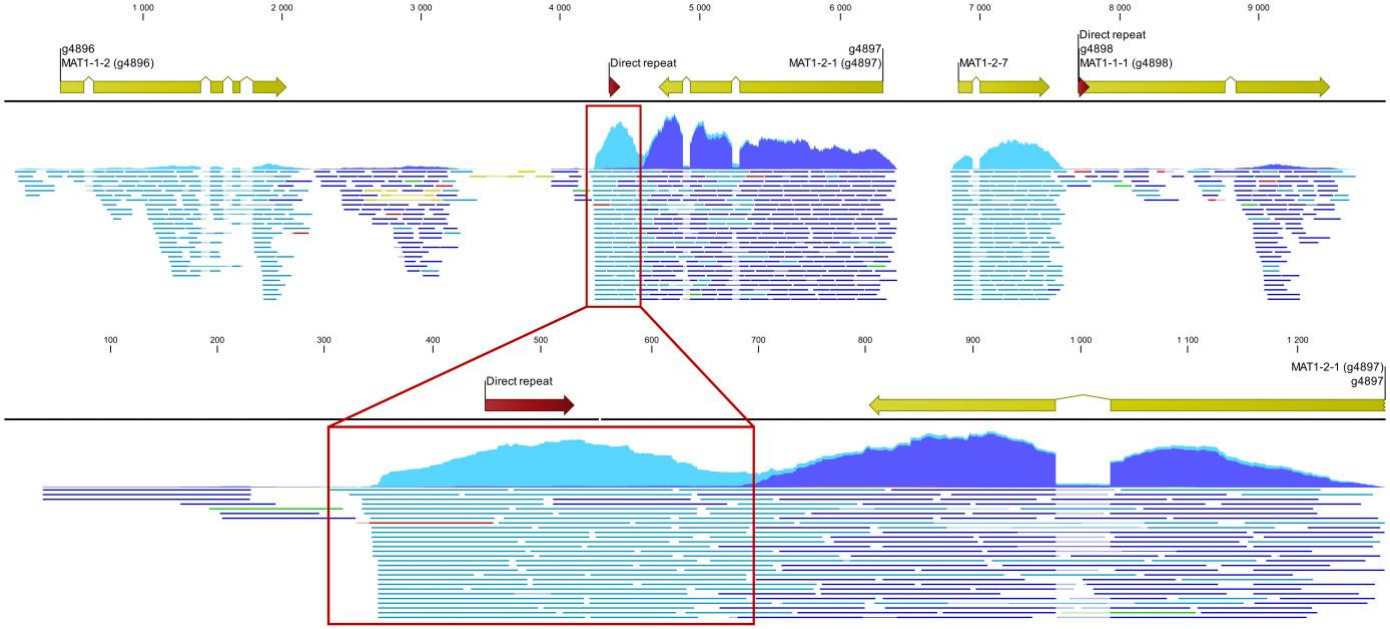
RNA reads from the MAT-2 self-sterile isolate mapped to the MAT-2 self-fertile *MAT1* locus configuration. Closer examination of the disconnected *MAT1-1-1* promoter region indicates transcripts that include this region as well as the direct repeat and some of the *MAT1-2* region. Yellow and red arrows indicate coding sequences and direct repeat sequences, respectively. Light blue regions on the graph indicate reads mapping to the positive strand, while dark blue regions in the graph show negative strand mapping. Pair-end reads mapping to the positive and negative strands are shown in light blue and dark blue, respectively, while single-end reads mapping to the positive and negative strands are indicated in green and red, respectively. Reads that could map to multiple regions are shown in yellow. Only the first 30 mapped rows are displayed, while the graph indicates overall read mapping counts.

RNA read mapping revealed transcriptional distinctions between the *MAT1* locus configurations, offering insights into locus structure and activity. This was the case in both the self-fertile and self-sterile *MAT1* locus versions (Fig. 3; Supplementary Fig. 3). Notably, reads mapping to a 5′ UTR was only detected for the *MAT1-1-1* gene in the MAT-1 self-sterile locus version, indicating a promoter sequence upstream of the single direct repeat. However, in the MAT-2 self-fertile version of the locus, this promoter and 5′ UTR are detached from *MAT1-1-1*, being separated by the *MAT1-*2 region. When MAT-2 self-fertile and MAT-2 self-sterile RNA reads were mapped to the MAT-2 self-fertile locus version, they aligned to the *MAT1-1-1* immediately upstream of the *MAT1-1-1* gene. Instead reads mapped to the promoter sequence and continued through the downstream direct repeat and approximately 170 bp into the *MAT1-2* region that is deleted during mating-type switching (Fig. 3). Although RNA reads of the MAT-2 self-sterile isolate mapped to the second exon of *MAT1-1-1*, it showed low mapping and the possible protein it could encode was 167 amino acids in length (compared to the full 579) and would not possess the conserved MAT α1 HMG-box domain.

#### Comparison of MAT-2 self-fertile and MAT-1 self-sterile isolates

**Fig. 4.**
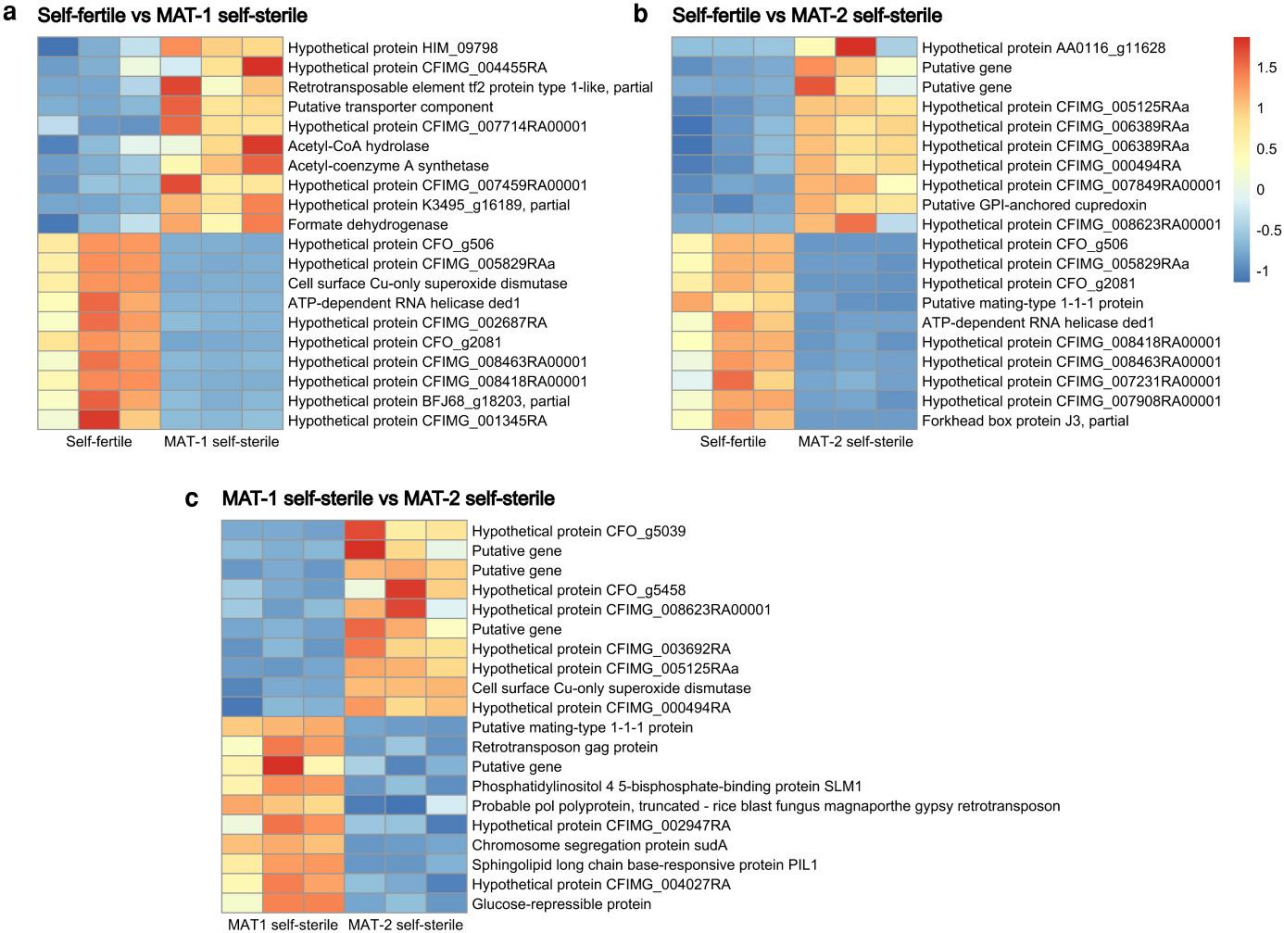
Heatmap showing the 10 genes with the highest positive log_2_ fold change for each isolate in 3 pairwise comparisons, including (a) MAT-2 self-fertile vs. MAT-1 self-sterile, (b) MAT-2 self-fertile vs. MAT-2 self-sterile, and (c) MAT-1 self-sterile vs. MAT-2 self-sterile isolates. Genes were selected from significantly differentially expressed sets (adjusted *P*-value < 0.05). Colors represent *Z*-score-scaled rlog-transformed expression values for each gene across samples, illustrating relative expression patterns. Genes labeled “putative gene” were not assigned a description during the Blast2GO analysis.

When comparing the expression data for the MAT-1 self-sterile to the MAT-2 self-fertile isolate, 371 genes were differentially expressed. Of these, 110 genes were upregulated in the self-sterile isolate, and 261 genes were upregulated in the self-fertile isolate. The top 10 highly upregulated genes in the self-sterile dataset included genes involved in acetyl-CoA catabolism and metabolism, as well as genes associated with transposable elements and viruses (Fig. 4a; Supplementary Table 4). The majority of these upregulated genes in the self-fertile isolate had no detectable conserved domain and was treated as hypothetical genes based on BLAST searches of the NCBI database (Supplementary Table 4).

Genes associated with the sexual cycle displayed variable expression patterns. The *MAT1-1-1* gene was expressed at similar levels in both the MAT-2 self-fertile and MAT-1 self-sterile isolates (Fig. 2a). The α-pheromone receptor was expressed only in the self-fertile isolate, whereas *MAT1-1-2* and the a-pheromone-receptor were expressed in both, but were upregulated in the self-fertile isolate. All pheromone processing genes and genes involved in the signal transduction pathway were expressed in both isolates, but no significant differential expression was noted.

**Fig. 5.**
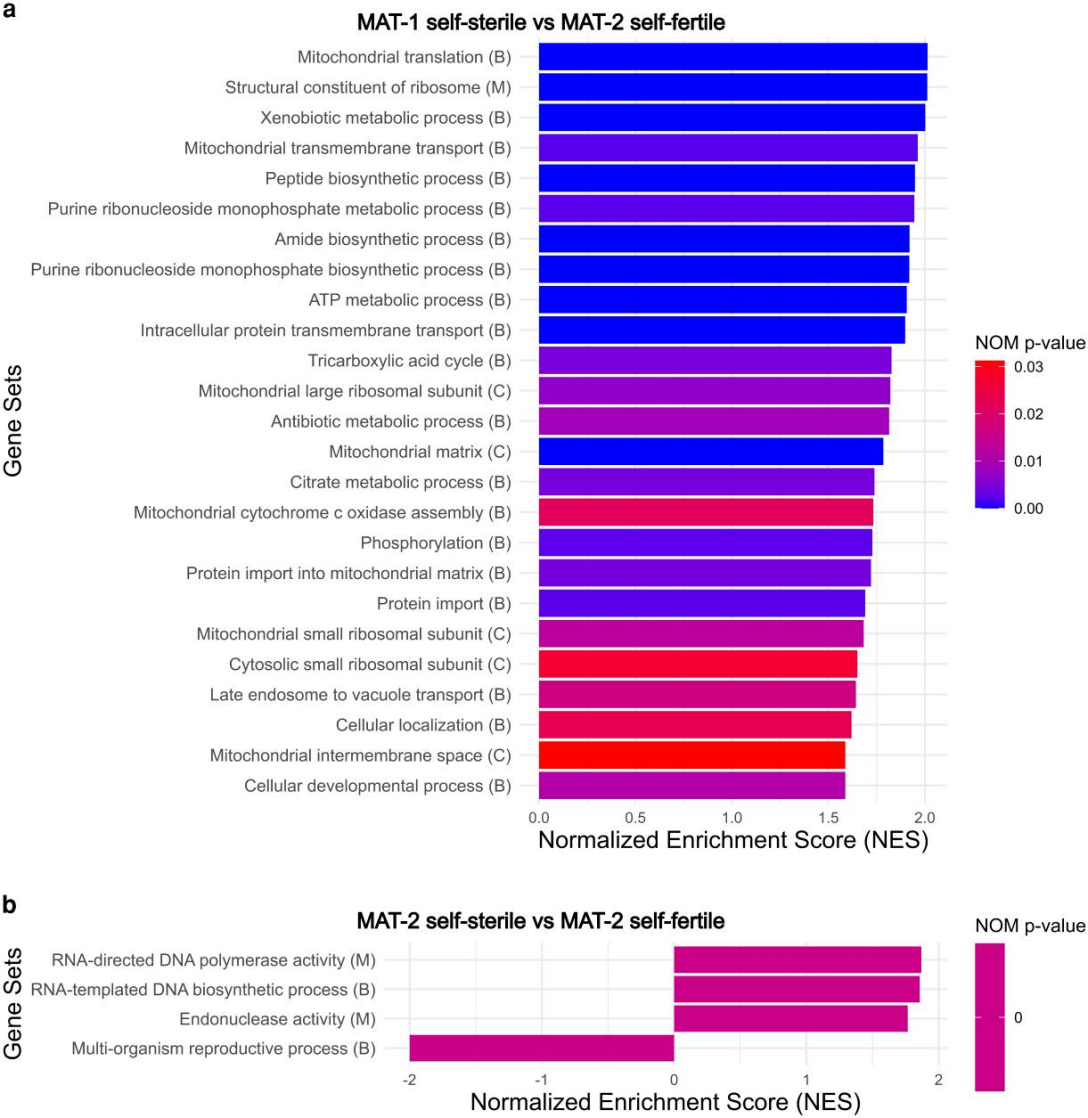
Gene set enrichment analysis (GSEA) of ranked differential expression results. Only gene sets meeting significance thresholds (FDR *P*-value < 0.25, |NES| > 1, NOM *P*-value < 0.25) are shown. Letters in brackets indicate the type of GO term: B = biological process, C = cellular component, M = molecular function. The normalized enrichment score (NES) indicates the magnitude and direction of enrichment along the ranked gene list (positive and negative values correspond to opposite ends of the ranking). The color scale represents the NOM *P*-value, providing estimates of the statistical significance of the enrichment score for a single gene set. Results from preranked GSEA for the self-fertile isolate when compared to (a) the MAT-1 self-sterile isolate and (b) the MAT-2 self-sterile isolate. Negative NES values indicate enrichment in the MAT-2 self-fertile isolate, while positive values show enrichment in the respective self-sterile individual.

Twenty-six gene sets were significantly enriched in the MAT-1 self-sterile isolate compared to the MAT-2 self-fertile (Fig. 5a; Supplementary Table 5). These gene sets were mostly related to the production and functioning of mitochondria. These also included gene sets involved in protein synthesis and movement, as well as antibiotic metabolism. No gene sets were significantly enriched in the self-fertile isolate.

#### Comparison of MAT-2 self-fertile and MAT-2 self-sterile isolates

A total of 1,034 genes were differentially expressed between the MAT-2 self-fertile and MAT-2 self-sterile isolates. In this comparison, more genes showed higher expression in the self-fertile isolate (573) than in the self-sterile isolate (461). The most strongly upregulated genes in the self-sterile isolate were largely annotated as hypothetical proteins with no conserved domains, with one exception containing a chromatin organization modifier domain (Fig. 4b; Supplementary Table 4). Among the top 10 upregulated genes in the self-fertile isolate were 2 transcription factors—*MAT1-1-1* and a forkhead box protein, while the rest were hypothetical genes, several lacking identifiable domains.

Genes linked to mating showed varied expression patterns between the 2 isolates. Both *MAT1-2* genes and the a-pheromone receptor were expressed at similar levels (Fig. 2b). In contrast, both *MAT1-1* genes and the α-pheromone receptor were significantly upregulated in the self-fertile isolate, with *MAT1-1-1* showing a 96-fold increase in expression relative to the self-sterile. Among pheromone processing genes, only *ram1*, which modifies the a-pheromone, was differentially expressed, with higher expression in the self-sterile isolate (Fig. 2b). Within the signal transduction pathway, *pp1* and *gnb1* were upregulated in the self-fertile isolate, while *gna1* was upregulated in the self-sterile; all other pathway components showed no significant differential expression.

Gene set enrichment analysis identified 4 significantly enriched gene sets in this comparison (Fig. 5b; Supplementary Table 6). Three of these were enriched in the self-sterile isolate and included functions related to RNA-to-DNA biosynthesis and endonuclease activity. The only gene set enriched in the self-fertile isolate was associated with reproductive processes in multicellular organisms.

#### Comparison of MAT-1 self-sterile and MAT-2 self-sterile isolates

Differential expression analysis between the MAT-1 and MAT-2 self-sterile isolates revealed 584 genes with significant changes in expression. Of these, 261 genes were upregulated in the MAT-1 self-sterile isolate, while 323 were upregulated in the MAT-2 self-sterile. Among the most highly expressed genes in the MAT-1 self-sterile were those associated with mating, transposable elements, and membrane or signaling functions (Fig. 4c). In contrast, the top 10 most upregulated genes in the MAT-2 self-sterile isolate were predominantly hypothetical or putative genes, lacking conserved domains and yielding no significant BLAST hits.

Although both isolates were self-sterile, key differences were observed in the expression of genes linked to the sexual cycle. The *MAT1-1-2* and a-pheromone-receptor genes were expressed at comparable levels in both isolates, whereas the α-pheromone receptor was exclusively expressed in the MAT-2 self-sterile isolate (Fig. 2c). The *MAT1-1-1* gene, expressed in both, exhibited a 140-fold higher expression in the MAT-1 self-sterile isolate. Most pheromone processing genes showed no significant differences in expression, except for *ram1*, which was upregulated in the MAT-2 self-sterile. A similar situation was seen in signal transduction pathway genes: *pp1* was more highly expressed in the MAT-1 self-sterile isolate, while *gna1* was upregulated in the MAT-2 self-sterile. No significantly enriched gene sets were identified in this comparison.

## Discussion

Mating-type genes are known as key regulators of the sexual cycle (Dyer et al. 2016; Wilson et al. 2021), but functional and chromatin immunoprecipitation studies highlight their broader roles in shaping fungal biology and development (Böhm et al. 2013; Becker et al. 2015; Doughan and Rollins 2016). In *Ceratocystis* species, unidirectional mating-type switching not only involves deletion of 2 *MAT1-2* genes but also appears to rearrange the locus, potentially affecting the expression of the *MAT1-1-1* gene (Wilken et al. 2024). While these genomic changes are known to directly change the sexual phenotype (Wilken et al. 2014; Yun et al. 2017), their downstream effects on gene regulation at a transcriptome-wide level, particularly in relation to self-fertile and self-sterile isolate types, remain poorly understood. In this study, we showed distinct transcriptomic signatures between 3 *C. albifundus* isolate types that were genetically identical, differing only at the *MAT1* locus. These included a MAT-1 self-sterile type (*MAT1-1* genes only), a MAT-2 self-sterile type (the literature-defined “MAT-2 self-fertile” locus with *MAT1-1* and *MAT1-2* genes), and a MAT-2 self-fertile heterokaryon containing both locus versions seen in the self-sterile isolates. There was clear differential expression of the *MAT* genes and associated components of the sexual signaling pathway, including pheromone precursors and their receptors. These findings underscore the central role of mating-type genes and their downstream networks in mediating broader transcriptional responses. They also suggest that unidirectional mating-type switching may have biological consequences that extend well beyond the regulation of sexual reproduction.

Previous research has proposed that unidirectional mating-type switching joins *MAT1-1-1* with its promoter, potentially affecting its expression profile (Wilken et al. 2024). In the self-fertile configuration, *MAT1-2* genes lie between *MAT1-1-1* and its promoter, blocking transcription. Switching removes this intervening region, restoring promoter proximity and enabling *MAT1-1-1* expression. This mechanism is similar to that in other fungi, where insertion of *MAT1-2* disrupts the *MAT1-1-1* coding sequence (Xu et al. 2016; Yun et al. 2017; Krämer et al. 2021). In our RNA-seq data, *MAT1-1-1* was expressed at similar levels in the MAT-2 self-fertile and MAT-1 self-sterile isolates, both of which possess the promoter-linked version of the gene. These results support the model that switching enables *MAT1-1-1* transcription (Wilken et al. 2024), facilitating the coexistence of nuclei expressing both core mating-type genes in a single individual. Since *MAT1-1-1* and *MAT1-2-1* are primarily responsible for sexual reproduction (Wilson et al. 2021), switching may serve as a mechanism to ensure their concurrent expression within a single individual.

Interestingly, partial transcripts from *MAT1-1-1* were detected in the MAT-2 self-sterile isolate (Fig. 3), despite this isolate carrying the self-fertile *MAT1* locus configuration (Wilken 2015; Engelbrecht and Harrington 2017). Transcript mapping showed high read density at the *MAT1-1-1* promoter but little beyond it, suggesting that transcription is initiated but prematurely terminated due to it continuing into intergenic sequence rather than *MAT1-1-1*. These results imply that while *MAT1-1-1* is constitutively induced, a functional transcript is only produced when it is connected to its promoter, as in the MAT-1 self-sterile locus. This appears similar to what has been found in *Ch. spinulosa*, where the full *MAT1-1-1* gene can only be expressed from the MAT-1 self-sterile locus (Yun et al. 2017). A small number of reads did map to the *MAT1-1-1* coding region, and the gene was detected as expressed. This could reflect a low level of successful switching, a view supported by minimal PCR amplification of the MAT-1 self-sterile locus in these isolates. Although a *MAT1* locus version allowing *MAT1-1-1* gene expression was therefore present, these isolates remained phenotypically self-sterile. Collectively, these results suggest that sexual reproduction in switching species may not depend only on the presence of both locus configurations, but also on their relative abundance within the mycelium.

Elevated expression of *MAT1-1-2* in self-fertile isolates points to a role beyond mating-type determination, potentially in later stages of sexual development. In this study, *MAT1-1-2* was expressed in all isolates but was notably upregulated in the MAT-2 self-fertile isolate. While its presence in self-sterile isolates could reflect a function in mating-type identity, increased expression in the self-fertile isolate, where sexual development is active, suggests a role beyond mate recognition. Secondary *MAT* genes like *MAT1-1-2* have been associated with postfertilization processes, such as fruiting body development (Arnaise et al. 2001; Rodenburg et al. 2018; Wilson et al. 2020). Uniquely, *MAT1-1-2* is the only *MAT* gene retained with its flanking regions in both self-fertile and self-sterile configurations across *Ceratocystidaceae* species capable of switching (Wilken et al. 2014). Its conserved genomic position and expression profile make the *MAT1-1-2* gene a strong candidate for functional studies aimed at understanding its role in the sexual cycle.

Pheromone-receptor expression in *C. albifundus* appears to be mating-type dependent and linked to sexual activity. While these receptors are typically expressed regardless of mating type (Kim and Borkovich 2004; Kim et al. 2012), some fungi do show mating-type-specific expression, where *MAT1-1* and *MAT1-2* genes control a- and α-pheromone receptor expression, respectively (Zhang et al. 1998; Shen et al. 1999; Wilson et al. 2020). In our study, expression of the α-pheromone receptor was absent in the MAT-1 self-sterile isolate, likely due to the loss of *MAT1-2* genes through mating-type switching. This suggests that α-pheromone receptor expression is dependent on *MAT1-2* genes. Both receptor genes were expressed in the MAT-2 self-fertile isolate and were generally upregulated relative to the self-sterile isolates, possibly reflecting an active role in the sexual cycle. These patterns are similar to those seen in *Huntiella moniliformis*, where α-pheromone receptor expression increases during active sexual reproduction (Wilson et al. 2018).

Pheromone gene expression did not match that of their cognate receptors, a discrepancy likely influenced by the timing of RNA sampling. Neither the a- nor α-pheromone genes were expressed in the MAT-2 self-fertile nor in either of the self-sterile isolates at the sampled time point. Although pheromone expression does not follow a strict temporal pattern, it is generally observed prior to or during sexual reproduction (Pöggeler 2000; Coppin et al. 2005; Kim and Borkovich 2006). In this study, a single time point was used to compare transcriptomic differences, with self-sterile isolates grown alone showing no signs of sexual development, while the self-fertile isolate was actively producing ascomata. Previous studies have shown that sexual reproduction can occur when MAT-1 and MAT-2 self-sterile isolates are paired (Harrington and McNew 1997; Engelbrecht and Harrington 2017), suggesting that pheromone expression may be triggered by interaction between compatible partners. The lack of expression in isolated self-sterile cultures highlights the need for time-course studies of mating interactions to better understand pheromone dynamics in switching-capable species.

While this study focused on mating-related gene expression, most differentially expressed genes were not directly linked to mate recognition or reproduction. This was reflected in gene set enrichment results, where only the “multiorganism reproductive process” category was enriched in the MAT-2 self-fertile isolate compared to its MAT-2 self-sterile counterpart. Given that the isolates are genetically identical apart from the *MAT1* locus, the widespread transcriptomic changes observed are striking. Many of the upregulated genes in the self-fertile isolate may contribute to sexual reproduction indirectly (Coppin et al. 1997; Wilson et al. 2019), as this phase involves extensive morphological, metabolic, and chemical changes that also occur during other stages of the fungal life cycle. Notably, the 2 self-sterile, vegetative isolates also differed substantially in gene expression, suggesting that the *MAT1* locus influences a wider range of biological processes. Previous studies have linked this region to traits such as conidial formation, growth, hyphal morphology, pathogenicity, and spore dimorphism (Lockhart et al. 2005; Zhan et al. 2007; Njambere et al. 2011; Böhm et al. 2013; Lee et al. 2015), emphasizing the need for functional studies of mating-type genes and locus architecture in pathogenic fungi like *C. albifundus*.

MAT-2 self-sterile isolates have previously been reported in *Ceratocystis* species (Harrington and McNew 1997), but the cause of this laboratory-observed phenotype remains unknown. Our comparison of MAT-2 self-sterile and MAT-2 self-fertile isolates did not reveal clear differences in the expression of core mating-related genes, with the exception of *MAT1-1-1* that requires mating-type switching to be expressed. The observed differences in other sex-associated genes were limited to genes with broader cellular functions (Xue et al. 2008; Aboelfotoh Hendy et al. 2019), making it unlikely that sterility can be explained by their altered transcription levels. In contrast, the self-sterile isolate showed enrichment of gene sets associated with RNA-directed DNA polymerase and endonuclease activity, consistent with increased retrotransposon activity. This pattern suggests that transposable element mobilization or broader genomic instability, rather than canonical mating pathway regulation, may contribute to the sterile phenotype. These findings remain speculative, but could provide an initial framework for future investigation into the mechanisms underlying sterility in these isolates.

## Supplementary Material

jkag128_Supplementary_Data

## Data Availability

Raw RNA reads generated in this study can be accessed on NCBI's Sequence Read Archive (SRA; accession PRJNA1424667), and the RNA analysis data can be accessed on NCBI's Gene Expression Omnibus (GEO; accession GSE330261). Supplemental material available at G3 online.
