# Supplementary material for: Mating-type locus structure affects gene expression in unidirectional mating-type switching fungi
Source: G3 (Bethesda). 2026 May 12;16(7):jkag128. doi: 10.1093/g3journal/jkag128 (PMC13334188; doi:10.1093/g3journal/jkag128)
Supplement: jkag128_Supplementary_Data [file jkag128_supplementary_data.zip › Supplemental_Material_G3-2026-406687.docx]

# Supplementary Materials

**Supplementary Tables**

**Supplementary table 1:** The primer sets used to screen for MAT-1 and MAT-2 self-sterile isolates.

| **Target** | **Name** | |  | **# on Suppl. Fig. 1** | **Sequence (5’-3’)** | **Amplicon size (bp)** | **Reference** |
| --- | --- | --- | --- | --- | --- | --- | --- |
| MAT-2 self-fertile locus | Albi unsw F |  |  | 1 | CGTTGTGTGAAGCTCAGGTG | 274 | This study |
|  | Albi unsw R |  |  | 2 | CGCTGGGCCATTATATCGTA |  |  |
| Post-deletion *MAT1* locus | Albi sw F |  |  | 3 | CTTTCGTCGACCCTTTTCTG | 569* | This study |
|  | Albi unsw R |  |  | 1 | CGCTGGGCCATTATATCGTA |  |  |
| *MAT1-1-2* | Albi  MAT1-2 F |  |  | 4 | ATAGCAAAGGTAATCGGTCT | 834 | Lee *et al.* (2015) |
|  | Albi  MAT1-2 R |  |  | 5 | GCCGTCGAAAGAATCCTA |  |  |
| *MAT1-2-1* | Albi  MAT2-1 F |  |  | 6 | CCCCTTCATTTGGCCCAT | 596 | Lee *et al.* (2015) |
|  | Albi  MAT2-1 R |  |  | 7 | CATCAAGTCTGTGCATCCA |  |  |

* Can produce an amplicon of 3 917 from the pre-deletion *MAT1* locus (see Suppl. Fig. 1)

**Supplementary Table 2:** Genes involved in the pheromone response pathway whose gene expression was closely analysed with the pheromone and pheromone-receptor, and *MAT* genes

| **Protein** | **Description** | |
| --- | --- | --- |
| **Processing of the α-pheromone protein** | |  |
| *kex1* | Pheromone-processing carboxypeptidase | |
| *kex2* | Pheromone-processing endoprotease | |
| *ste13* | Dipeptidyl aminopeptidase | |
| **Processing of the a-pheromone protein** | |  |
| *ram1* | Protein farnesyltransferase | |
| *ram2* | Protein farnesyltransferase | |
| *rce1* | CAAX prenyl protease | |
| *ste6* | ATP-binding cassette a-factor transporter | |
| *ste14* | Farnesyl cysteine-carboxyl methyltransferase | |
| *ste24* | CAAX prenyl protease | |
| *axl1* | Endoprotease | |
| **Signal transduction pathway** | |  |
| *gna1* | G-protein alpha-1 subunit | |
| *gna2* | G-protein alpha-2 subunit | |
| *gnb1* | G-protein beta subunit | |
| *gng1* | G-protein gamma subunit | |
| *mekk3 ^*^* | Mitogen activated protein kinase kinase kinase | |
| *mek2* | Mitogen activated protein kinase kinase 2 | |
| *mak2* | Mitogen-activated protein kinase-2 | |
| *pp1* | Serine/threonine-protein phosphatase | |

* Also referred to as *nrc1*

**Supplementary table 3:** Details on RNA-seq data

| **Samples** | **Concentration (ng/ul)** | **Raw reads (mil)** | **Trimmed reads (mil)** | **Reads mapped in pairs and broken pairs (%)** | **Transcripts (mil)** |
| --- | --- | --- | --- | --- | --- |
| MAT-2 self-fertile rep 1 | 188.3 | 48.9 | 48.9 | 87.90 | 10.5 |
| MAT-2 self-fertile rep 2 | 435.5 | 55.1 | 55.1 | 89.93 | 12.1 |
| MAT-2 self-fertile rep 3 | 765.0 | 37.4 | 37.4 | 87.92 | 8.0 |
| MAT-1 self-sterile rep 1 | 681.9 | 43.6 | 43.6 | 90.11 | 9.6 |
| MAT-1 self-sterile rep 2 | 976.3 | 44.2 | 44.2 | 90.75 | 10.0 |
| MAT-1 self-sterile rep 3 | 846.8 | 46.3 | 46.3 | 85.50 | 9.2 |
| MAT-2 self-sterile rep 1 | 659.4 | 33.6 | 33.6 | 91.07 | 7.1 |
| MAT-2 self-sterile rep 2 | 790.2 | 35.5 | 33.5 | 90.62 | 7.2 |
| MAT-2 self-sterile rep 3 | 1 489.0 | 37.7 | 37.7 | 89.60 | 8.0 |

**Supplementary table 4:** Hypothetical genes that were part of the top ten differentially expressed genes for each isolate in each comparison for which a conserved domain could be identified

| **Isolate in which gene was upregulated** | **Hypothetical protein** | **Conserved domain(s)** |
| --- | --- | --- |
| **Self-fertile vs. MAT-1 self-sterile** | | |
| MAT-1 self-sterile | Hypothetical protein HIM_09798 | Reverse transcriptase (RNA-dependent DNA polymerase)  RNase H |
|  | Hypothetical protein CFIMG_007714RA00001 | Integrase zinc binding domain |
|  | Hypothetical protein K3495_g16189, partial | RNase H-like domain found in reverse transcriptase  RNase H |
| Self-fertile | Hypothetical protein CFIMG_002687RA | Zinc-finger of C2H2 type |
|  | Hypothetical protein CFIMG_008418RA00001 | Phosphatidate phosphatase APP1  Catalytic domain |
|  | Hypothetical protein BFJ68_g18203, partial | DDE superfamily endonuclease |
| **Self-fertile vs. MAT-2 self-sterile** | | |
| MAT-2 self-sterile | Hypothetical protein AA0116_g11628 | Chromo (CHRromatin Organisation MOdifier) domain |
| Self-fertile | Hypothetical protein CFIMG_008418RA00001 | Phosphatidate phosphatase APP1  Catalytic domain |
|  | Hypothetical protein CFIMG_007231RA00001 | Helix-hairpin-helix domain |

**Supplementary Figures**


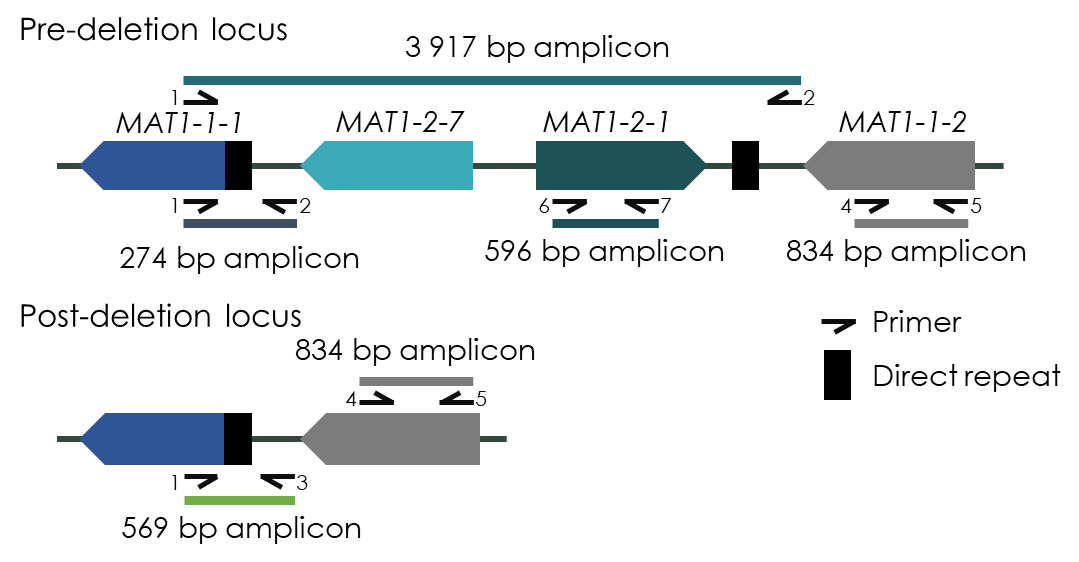


**Supplementary Figure 1:** A visual representation of the two versions of *MAT1* locus with the binding sites and expected amplicons from the primer sets used to determine the fertility type of an isolate. This figure is not drawn to scale. Primers used can be found in Suppl. Table 1.

**
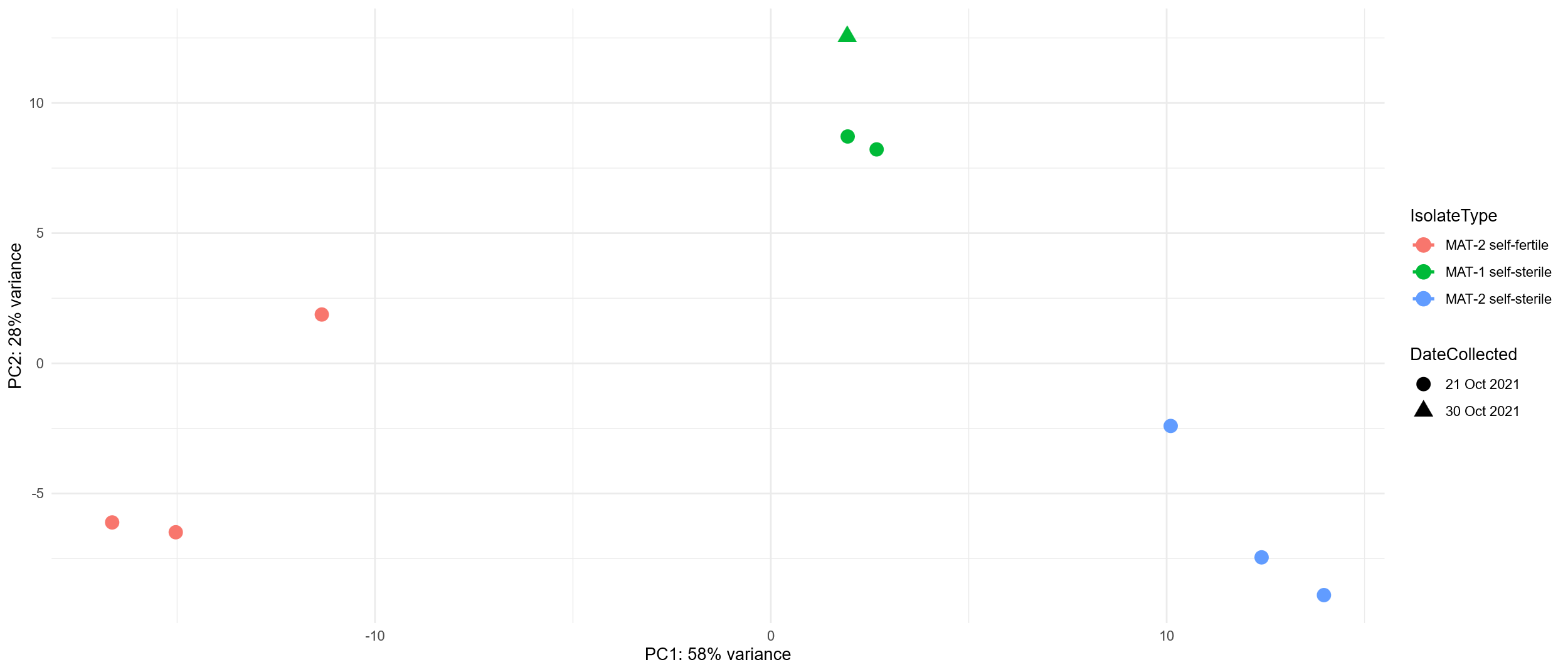
**

**Supplementary Figure 2:** PCA plots of regularised-logarithmic (rlog) transformation data sets.


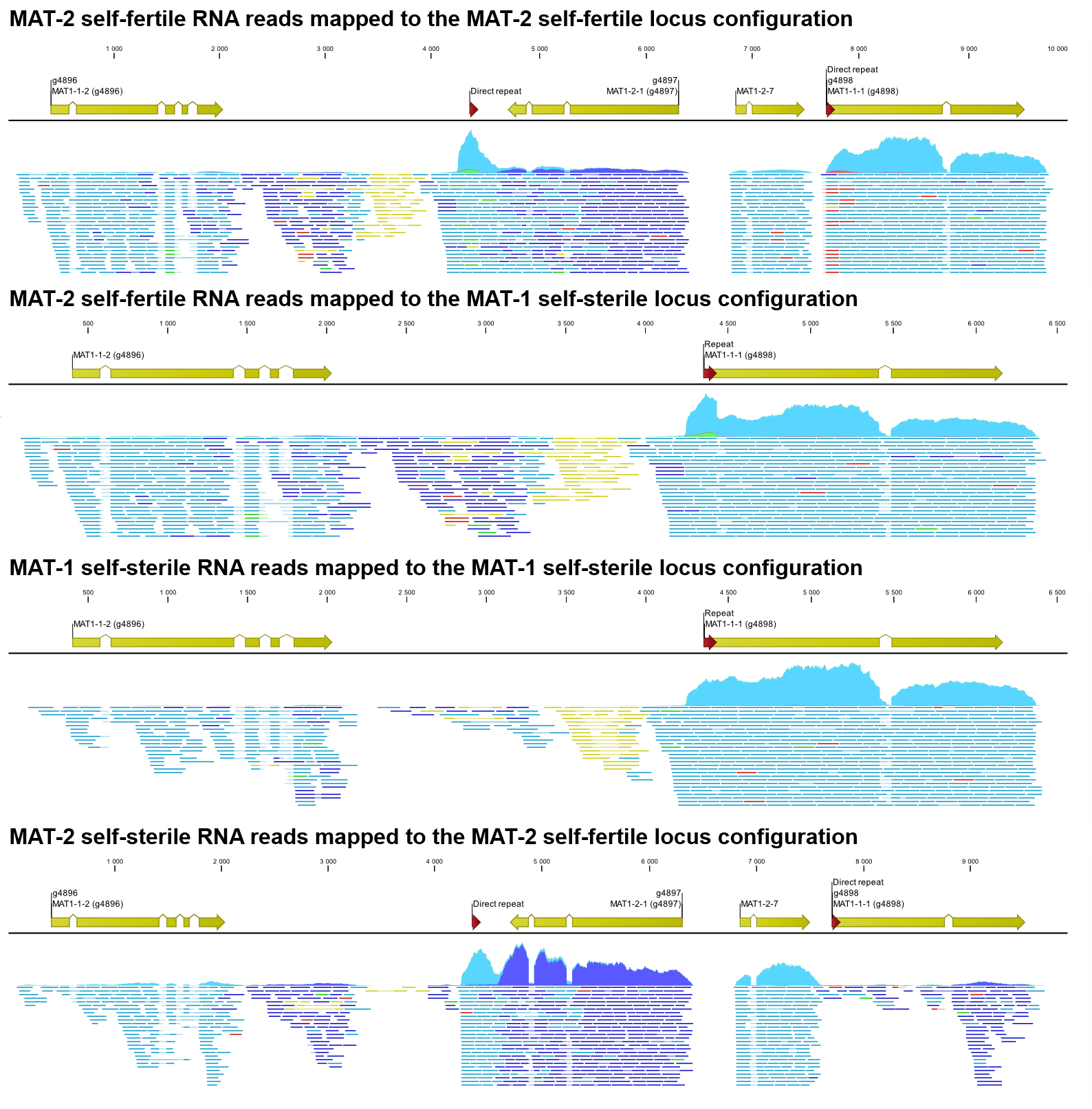


**Supplementary Figure 3:** RNA reads mapped to the MAT-2 self-fertile and MAT-1 self-sterile mating-type locus configuration. Yellow arrows indicate genes and red arrows indicate direct repeat regions. Pair-end reads mapping to the positive and negative strands are shown in light blue and dark blue respectively, while single-end reads mapping to the positive and negative strand are indicated in green and red respectively, and reads mapping to multiple regions are shown in yellow. Only the first 30 rows of read mapping are displayed here, with the graph indicating overall read counts.
